# Supplementary material for: A Systems Biology Approach for Personalized Medicine in Refractory Epilepsy
Source: Int J Mol Sci. 2019 Jul 30;20(15):3717. doi: 10.3390/ijms20153717 (PMC6695675; doi:10.3390/ijms20153717)
Supplement: Supplementary file 1 [file ijms-20-03717-s001.pdf]

Table 1 PK and PD alterated genes in refractory epilepsy

Gene	Alterations	Product	References	Possible mechanisms of drug refractoriness
ABCB1 (MIM171050)	C3435T	ATP-binding cassette (ABC) transporter	[32-37]	Overexpression or increased activity of efflux transporter in the blood-brain barrier enhanced drug extrusion from target site
	T1236C		[36, 38, 39]	
	G2677T		[36-38]	
	G2677A		[36, 38]	
ABCC2 (MIM601107)	ABCC2 - 24T		[47]	
CYP3A4 (MIM124010)	CYP3A4*1B	Cytochrome P450 3A4 enzyme	[49, 52-54]	Alterations in metabolic activity
CYP2C9 (MIM601130)	CYP2C9*3	Cytochrome P450 2C9 enzyme	[48, 49, 54]	
	CYP2C9*3/*3			
SCN1A (MIM182389)	SCN1A IVS5-91 G>A	Voltage-gate Na^+ channel α1 subunit	[72]	Structure alteration and reduction of sensitivity to the drug
	rs6730344A/C		[74]	
	rs6732655A/T		[74]	
	rs10167228A/T		[74]	
SCN2A (MIM182390)	c.56 G→A	Voltage-gate Na^+ channel α2 subunit	[71]	
	c.R19K		[70]	
	IVS7-32A>G		[69]	
GABRA1 (MIM137160)	IVS11 + 15 A> G	GABA$_A$ receptor α1 subunit	[84]	Aberrant formation of the drugs-binding pocket
	rs6883877		[85]	
GABRA2 (MIM137140)	rs511310	GABA$_A$ receptor α2 subunit	[85]	
GABRA3 (MIM305660)	rs4828696	GABA$_A$ receptor α3 subunit	[85]	
GRIN2A (MIM138253)	L812M	NMDA receptor GluN2A subunit	[92]	Greater glutamatergic activity and reduced Mg^{2+} negative modulation
	M817V		[91]	
